# Preserving Fertility After Bilateral Testicular Gunshot Injury: A Case Report

**DOI:** 10.1155/cris/1140740

**Published:** 2026-06-19

**Authors:** Rashad Sholan, Rufat Aliyev, Seymur Karimov

**Affiliations:** ^1^ Scientific Research Center, State Security Service Military Hospital, Baku, Azerbaijan; ^2^ Scientific Research Center, Azerbaijan Medical University, Baku, Azerbaijan, amu.edu.az; ^3^ Laboratory of Immunophysiology and Experimental Transplantology, Institute for the Study of Living Systems, Ministry of Science and Education, Baku, Azerbaijan; ^4^ Department of Kidney Diseases and Organ Transplantation, State Security Service Military Hospital, Baku, Azerbaijan

**Keywords:** fertility preservation, gunshot wounds, orchiectomy, scrotal trauma, testicular injuries

## Abstract

Penetrating scrotal trauma from gunshot injuries is rare but can severely impact fertility and endocrine function. We report a 32‐year‐old male with bilateral testicular injury managed with left partial orchiectomy and right testicular repair. Viable tissue was preserved without grafts, and perfusion was confirmed intraoperatively. Postoperative recovery was uneventful, with Doppler ultrasonography showing bilateral viability. Initial severe asthenozoospermia improved markedly over 2 years. This case underscores the potential for testicular salvage and fertility preservation in severe bilateral injuries, highlighting the value of prompt, tailored surgical intervention.

## 1. Introduction

Penetrating injuries to the external genitalia are among the most complex forms of genitourinary trauma, often resulting in significant physical and psychological morbidity. Gunshot wounds to the scrotum and testes, although relatively rare in civilian populations, typically require urgent surgical intervention due to the high risk of infection, tissue devitalization, and impaired reproductive and endocrine function [1, 2]. Bilateral testicular injuries, in particular, pose a substantial threat to fertility and hormonal balance, with many cases necessitating orchiectomy and leading to permanent hypogonadism or infertility [3].

The clinical approach to such injuries emphasizes prompt hemostasis, debridement of necrotic tissue, and, whenever feasible, preservation of viable testicular parenchyma [4]. While unilateral damage may be managed with acceptable long‐term outcomes, bilateral involvement raises critical challenges in preserving both testicular function and fertility potential.

This report presents the case of a 32‐year‐old male with bilateral testicular injury due to gunshot trauma, managed with partial orchiectomy and conservative repair. The aim is to highlight the potential for testicular salvage and the restoration of fertility through timely and meticulous surgical management, emphasizing the importance of individualized care in severe genital trauma.

## 2. Case Presentation

A 32‐year‐old male patient was admitted to the emergency department 2 h after sustaining a gunshot injury to the genital region. On physical examination, he was alert, hemodynamically stable, and complained of severe scrotal pain. Local inspection revealed a laceration of the left hemiscrotum with protrusion of testicular tissue and swelling of the right testis. Initial scrotal ultrasonography with color Doppler imaging showed edema and decreased vascularity in the right testis and spermatic cord, while the left testis demonstrated disrupted parenchymal architecture and absent vascular flow. Bilateral hematoma and scrotal edema were also noted.

**Figure 1:**
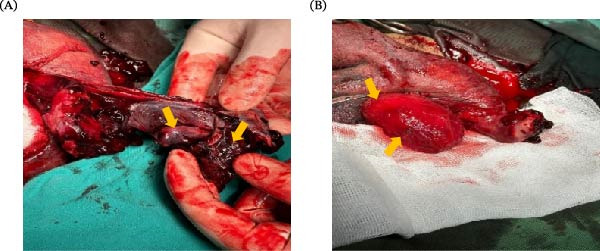
Intraoperative images demonstrating the extent of bilateral testicular injury: (A) the left testis and epididymis (yellow arrows) showing extensive laceration and devitalized tissue and (B) the right testis (yellow arrows) with preserved viable parenchyma following debridement.

The patient was promptly taken to the operating room for scrotal exploration. Intraoperatively, ~70% of the left testicular tissue was found to be devitalized, and partial rupture of the right spermatic cord was observed (Figure 1). A partial orchiectomy was performed on the left side, preserving the viable segment. On the right, the lacerated testicular tissue was sutured, and perfusion was improved using warm saline irrigation and local papaverine application. Hemostasis was achieved, and both testes were repositioned and fixed within the scrotum. The early postoperative course was uneventful. On postoperative Day 7, Doppler ultrasonography confirmed preserved vascularity in the right testis. However, the left testis appeared smaller in size with reduced echogenicity and absent vascularity. By postoperative Week 7, the right testis remained normal, while the left testis showed signs of fibrosis with heterogeneous echotexture and minimal vascular flow.

Serial semen analyses were performed to monitor fertility potential. At postoperative Week 7, the patient was found to have severe asthenozoospermia (volume: 6 mL; concentration: 18 million/mL; motility: 0%). At 4 months, mild improvement was observed (volume: 7 mL; concentration: 21 million/mL; motility: 5%). By the second postoperative year, semen parameters had significantly improved, with a volume of 7 mL, sperm concentration of 22 million/mL, and motility of 21%, indicating substantial recovery.

**Figure 2:**
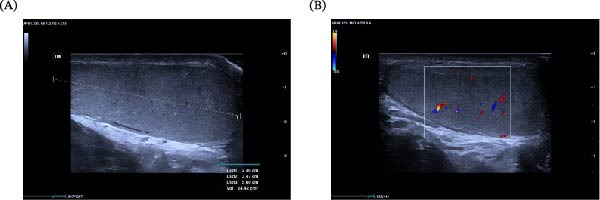
Long‐term follow‐up scrotal ultrasonography and color Doppler imaging. (A) B‐mode ultrasound demonstrating the normal dimensions and homogeneous parenchymal echotexture of the preserved right testis. (B) Color Doppler imaging confirming normal vascularity and robust perfusion within the right testicular parenchyma.

A supportive medical regimen consisting of daily supplementation with L‐carnitine 1000 mg, vitamin E 400 IU, Zinc 15 mg, and selenium 50 mcg was initiated after the first semen analysis and continued for a duration of 6 months. The patient continues regular outpatient follow‐up. To evaluate the long‐term outcomes of the testis‐sparing surgery, the patient was recently recalled for an updated follow‐up at the third postoperative year. Follow‐up scrotal ultrasonography with color Doppler imaging revealed an absent left testis with expected postoperative changes. However, the right testis demonstrated normal dimensions (35 mm × 24 mm × 56 mm, volume = 24.43 cc), a homogeneous parenchymal echotexture, and completely normal vascularization (Figure 2). There was no evidence of varicocele or reflux during the Valsalva maneuver. Concurrently, the most recent semen analysis demonstrated a remarkable continuous improvement, revealing a volume of 5.0 mL, a sperm concentration of 36 million/mL, a total sperm count of 180 million, total progressive motility (A + B) of 45%, and normal morphology of 24%. These findings confirmed the complete restoration of fertility potential. In addition to the recovery of exocrine function, the patient’s endocrine and sexual functions were systematically evaluated. Throughout the follow‐up period, the patient reported normal libido, preserved erectile function, and an absence of any sexual complaints. Concurrently, his specific serum hormone panel remained consistently within the normal physiological ranges (most recently, total testosterone: 480 ng/dL, FSH: 4.2 mIU/mL, and LH: 5.1 mIU/mL), confirming that the surviving right testicular parenchyma successfully maintained full endocrine and sexual health.

## 3. Discussion

Penetrating trauma to the male external genitalia, particularly from gunshot wounds, presents complex challenges in both functional and psychological domains. Although testicular salvage remains a primary goal, bilateral involvement often results in orchiectomy and irreversible loss of fertility or endocrine function [3, 5, 6]. The present case underscores the potential for testicular preservation and fertility recovery through prompt and meticulous surgical intervention, even in the setting of severe bilateral testicular injury.

Our approach aligns with recommendations from multiple retrospective series advocating early scrotal exploration, debridement of nonviable tissue, and primary repair when feasible [4, 7, 8]. Simhan et al. [5] reported a 52% testicular salvage rate among 50 injured testes, emphasizing that immediate exploration was crucial for favorable outcomes. Similarly, Phonsombat et al. [9] highlighted a 75% salvage rate for gunshot‐related testicular injuries, substantially higher than that observed in other mechanisms such as stab wounds.

While our patient underwent a left partial orchiectomy due to extensive parenchymal damage, viable tissue was preserved, and right‐sided repair was successfully performed, consistent with findings from Ferguson and Brandes [10], who demonstrated that even severely ruptured testes can be reconstructed when vascular integrity is maintained. Notably, synthetic grafts such as polytetrafluoroethylene have been associated with increased infection risk, whereas autologous tunica vaginalis remains a preferred material for albugineal reconstruction [10]. In our case, primary closure was achieved without grafting, and testicular perfusion was supported intraoperatively with warm irrigation and papaverine. However, it is important to acknowledge that despite the initial attempt to preserve the left testicular remnant, postoperative imaging revealed progressive fibrosis and eventual atrophy. Consequently, the left testis did not retain meaningful exocrine or endocrine function over time. The remarkable functional recovery and improvement in semen parameters observed in our patient were primarily, if not entirely, derived from the successfully repaired contralateral (right) testis. This physiological outcome highlights that in severe bilateral trauma, meticulous reconstruction and preservation of even a single viable testis can be sufficient to rescue the patient’s long‐term fertility.

Preserved endocrine function is frequently reported after unilateral orchiectomy; however, bilateral involvement poses a higher risk for hypogonadism and infertility [5, 11]. Although initial semen analysis revealed severe asthenozoospermia, our patient demonstrated a continuous and significant improvement, eventually achieving fully normalized sperm parameters (including a total progressive motility of 45%) in the long‐term follow‐up. This is encouraging, as scrotal firearm injuries have been linked to exocrine dysfunction due to both direct trauma and autoimmune responses following blood–testis barrier disruption [4]. Previous reports have largely focused on unilateral trauma or described cases necessitating total orchiectomy, with fewer examples highlighting successful fertility preservation in bilateral injuries. Kadouri et al. [4] presented a case of unilateral orchidectomy and contralateral repair, in which the patient experienced residual oligoasthenoteratozoospermia despite preserved testosterone levels. Our case expands upon these findings by demonstrating complete functional recovery with preserved fertility potential following bilateral intervention. Our findings further support the utility of early Doppler ultrasonography in guiding surgical decisions. As shown by Learch et al. [12], sonographic evidence of heterogeneous echotexture and contour disruption reliably predicts testicular rupture. In our case, preoperative imaging accurately correlated with intraoperative findings and postoperative follow‐up confirmed bilateral testicular viability.

When evaluating the potential for fertility recovery following severe scrotal trauma, several prognostic factors must be considered. First, the mechanism and precise anatomical location of the injury are pivotal; interestingly, previous reports indicate that penetrating gunshot wounds can yield higher testicular salvage rates compared to stab wounds, provided that the hilar vasculature and spermatic cord remain intact [9]. Second, the percentage of preserved viable testicular volume is a critical determinant of long‐term exocrine function, underscoring the absolute necessity of conservative debridement. Third, patient age plays a vital role, as younger patients generally possess a more robust capacity for cellular regeneration and compensatory spermatogenesis. Finally, the time elapsed until surgical intervention is paramount; prompt exploration minimizes ischemic time and limits the disruption of the blood‐testis barrier, thereby mitigating the risk of anti‐sperm antibody formation and subsequent autoimmune infertility [13, 14]. Importantly, responding to gunshot injuries requires specific surgical considerations distinct from blunt or stab traumas. The high‐velocity nature of firearms often creates a “blast effect,” resulting in microvascular damage and delayed tissue necrosis far beyond the visible bullet tract. Therefore, intraoperative management must prioritize copious irrigation and aggressive debridement of all contaminated and devitalized tissues to prevent severe postoperative infections and limit the release of testicular antigens that could trigger an autoimmune response. Furthermore, surgeons must strictly avoid synthetic materials for reconstruction due to the high infection risk and meticulously trace the bullet trajectory to rule out occult injuries to the urethra, contralateral structures, and adjacent pelvic organs.

In conclusion, this case emphasizes the importance of individualized surgical management, including selective partial orchiectomy and testicular repair, to maximize the likelihood of preserving endocrine and reproductive function. The restoration of normal semen parameters following timely intervention illustrates the potential for fertility salvage even in severe bilateral testicular trauma. Further prospective studies are needed to refine surgical techniques and long‐term fertility support in such patients.

## Author Contributions

**Rashad Sholan**: conceptualization, investigation, data curation, writing – original draft, writing – review and editing, supervision. **Rufat Aliyev**: surgical management, investigation, validation, writing – review and editing. **Seymur Karimov**: patient follow‐up, data collection, visualization, writing – review and editing.

## Funding

The authors received no financial support for this article.

## Disclosure

All content was critically reviewed, verified, and approved by the authors prior to submission. A preliminary version of this work was presented as a poster at the 34th National Urology Congress, October 23–26, 2025, Cyprus.

## Consent

Written informed consent was obtained from the patient for the publication of this case report and any accompanying images.

## Conflicts of Interest

The authors declare no conflicts of interest.

## Data Availability

The data that support the findings of this study are available from the corresponding author upon reasonable request.
